# Burying Alive

**Published:** 1876-03

**Authors:** 


### BURYING ALIVE.

INDIVIDUALS have been buried before life was extinct. Authentic cases are given where persons have been perfectly powerless, every muscle of the body paralyzed; but the hearing, the last sense to die, was in its integrity, and made them conscious, as far as sound was concerned, of all that was going on around them.

A physician was recently married in this city, and died within half an hour at noon-day. The body was removed to a distant county, and when the coffin-lid was removed to enable sorrowing friends to take a last look, a strange change passed over his face.

Many persons have felt, at night, as if some terrible danger were impending, but without any more power to move a muscle than to move a world. We know, too, what a fearful noise such persons make when a friendly shake starts up the stagnant blood, giving instant and most grateful relief.

